# Whether stoma support rods have application value in loop enterostomy: a systematic review and meta-analysis

**DOI:** 10.1186/s12957-020-02029-w

**Published:** 2020-10-22

**Authors:** Rui Du, Jiajie Zhou, Feng Wang, Dongliang Li, Guifan Tong, Xu Ding, Wei Wang, Daorong Wang

**Affiliations:** 1 Graduate School, Dalian Medical University, West Section of Lvshun South Road No. 9, China, Dalian, 116044 China; 2 Graduate School, Medical College of Yangzhou University, Huaihai Road No.7, Yangzhou, 225001 China; 3 Department of General Surgery, Northern Jiangsu People’s Hospital, Nantong Road No.98, Yangzhou, 225001 China

**Keywords:** Loop enterostomy, Colorectal cancer, Stoma support rods, Stoma retraction, Meta-analysis

## Abstract

**Purpose:**

The purpose of the systematic review and meta-analysis is to analyze the application value of the stoma support rods in loop enterostomy.

**Methods:**

The studies on the application of stoma rods in loop enterostomy published from January 2000 to January 2020 were searched in the databases of PubMed, Embase, Cochrane library, and Clinical trials. All randomized controlled trials (RCTs) and cohort studies that observed the value of stoma rods were included according to inclusion criteria. The RevMan5.3 software was used for statistical analysis.

**Results:**

A total of 1131 patients with loop enterostomy in six studies were included in this study; there were 569 cases in the experimental group and 562 cases in the control group. All six studies analyzed the effect of support rods on the incidence of stoma retraction; the meta-analysis showed that in a total of 32 patients, stoma retraction occurred, with a total incidence of about 2.8% in 1131 patients. The incidence of stoma retraction in the rod group was not significantly lower than that in the non-rod group, and the difference was not statistically significant (OR = 0.65, 95% CI 0.32~1.32, *I*^2^ = 0%, *P* = 0.23), and the studies were homogeneous. The incidences of stoma necrosis (OR = 6.41, 95% CI 2.22~18.55, *I*^2^ = 0%, *P* = 0.0006), peristomal dermatitis (OR = 2.93, 95% CI 2.01~4.27, *I*^2^ = 0%, *P* < 0.00001), and mucocutaneous separation (OR = 2.14, 95% CI 1.03~4.47, *I*^2^ = 0%, *P* = 0.04) were significantly increased in the rod group.

**Conclusions:**

It is not recommended to routinely use stoma support rods in the clinical practice.

## Introduction

Enterostomy is a common operation in colorectal surgery, which refers to leading a section of intestinal tube to the body surface to make an opening through surgery, to achieve the purpose of diverting intestinal contents. According to Chinese statistics [1], the total number of enterostomy patients in China has exceeded one million, and this number is still growing at the rate of 100,000 per year, of which malignant tumor patients are the main stoma patients, while colorectal cancer ranks first. Furthermore, the global incidence of colorectal cancer is increasing year by year [2], and the number of patients undergoing enterostomy continues to increase. At present, surgical resection is still the main treatment for colorectal cancer, and the occurrence of postoperative anastomotic leakage is a difficult problem for surgeons. To reduce the occurrence of postoperative anastomotic leakage and reduce the clinical symptoms caused by anastomotic leakage, for patients with low tumor location and high risk of postoperative anastomotic leakage, most operators will choose preventive loop enterostomy after radical tumor resection [3].

The retraction of the stoma is one of the serious complications after loop enterostomy, which means that the level of the intestinal mucosa is lower than that of the skin. It often makes the stoma care device unable to seal, thus increasing the leakage of intestinal contents and surrounding skin irritation. In more severe cases, stoma retraction can lead to separation of skin and mucosa, and even necrosis, which in turn leads to subcutaneous, subfascial, or abdominal contamination and systemic sepsis. In the later stage, the hyperplasia of skin or granulation tissue around the stoma can lead to stoma stenosis and obstruction, and severe cases often need to be treated again. Fear of retraction and subsequent peritonitis was the main driving force for the use of the stoma rod; most surgeons usually place a self-made or special rod-shaped stent in enterostomy to support the intestinal tube, but its effectiveness has not been confirmed. Although some studies have shown that the application of support rods can reduce the stoma retraction rate to 2% [4, 5], they only include a small number of patients, and there is no big data study to prove that the application of support rod can reduce the incidence of stoma retraction. Therefore, this systematic review and meta-analysis attempts to determine whether support rods can reduce the incidence of complications in enterostomy. Besides, the purpose of this study is to determine whether there is evidence-based justification for the routine use of stoma support rods.

## Methods

### Study search and selection

This systematic review was conducted following the Preferred Reporting Items for Systematic Reviews and Meta-Analyses (PRISMA) guidelines [6]. We searched the three foreign language databases MEDLINE, Embase, Cochrane Library, and Clinical trials, published from January 2000 to January 2020, and collected comprehensive RCTs or cohort studies on the effect of enterostomy support rods on the incidence of stoma retraction and other stoma-related complications. The search terms are “enterostomy[Mesh] or colostomy[Mesh] or ileostom[Mesh] or colon ostomy[Title/Abstract] or ileum ostomy[Title/Abstract] or colon stoma[Title/Abstract] or ileum stoma[Title/Abstract]” + “rod[Title/Abstract] or support[Title/Abstract] or holder[Title/Abstract] or stand[Title/Abstract]” + “Humans[Mesh]not Animals[Mesh]”. We further manually searched some references from studies we retrieved to supplement the results of computer searches, and the selection of the studies were carried out by three independent reviewers (R.D, J.Z, and F.W), if necessary, we also contacted the original author by phone or email to ask for more information. A meeting will be held to discuss the feasibility of the study when the three independent reviewers disagree with each other in the selection process.

### Inclusion and exclusion criteria

Inclusion criteria based on PICOS principles are as follows: (1) studies of patients with loop enterostomy over 18 years old, (2) compared the use of stoma support rods with non-rods in loop enterostomy, (3) both included loop ileostomy and colostomy, (4) revealed adequate data of the incidence of stoma retraction and other stoma complications, (5) any randomized controlled trials (RCTs) and cohort studies.

Exclusion criteria are as follows: (1) studies that do not meet the inclusion criteria; (2) reviews, case reports, comments, and conference abstracts; (3) studies whose important data are incomplete and cannot be obtained by other means; (4) repeatedly published studies; (5) studies with doubtful research methods; (6) studies with unclear effect judgment indicators.

### Data collection

Two evaluators (R.D and J.Z) read the full text of the literature independently, extracted the relevant information, and compared the final data, and the dispute was decided by the third evaluator (F.W). Literature extraction includes the following contents: (1) the general information included in the literature: the title of the literature, the author, the year of publication, the country of the study, the time of the study, the sample size of the experimental group and the control group, inclusion and exclusion criteria; (2) research quality evaluation: random allocation method, allocation scheme hiding, blind method; (3) intervention and control measures: the type of colostomy support rod and the indwelling time of support rod; (4) outcome: stoma retraction, stoma necrosis, peristomal dermatitis, mucocutaneous separation, peristomal infection, the length of placement of stoma rod, hospital stay, etc.

### Assessment for risk of bias

All studies were assessed for risk of bias using the bias risk assessment method [7] recommended by the Cochrane system evaluator manual. Two review authors (R.D and J.Z) independently assessed the risk of bias in included trials using the tool of the Cochrane Collaboration for assessing risk of bias, as described in the Cochrane Handbook for Systematic Reviews of Interventions. Each item was rated as “Low risk,” “Unclear risk,” or “High risk” of bias. We resolved discrepancies through discussion or adjudication by a third review author (F.W or D.L).

We used the Newcastle-Ottawa scale [8] to evaluate the quality of cohort studies, which consisted of three categories (selection, comparability, and outcome) and eight elements with a maximum score of nine, which was used to evaluate the quality of enrolled observational research. Studies graded with 7 or above were considered as high quality. The above assessment was performed independently by three authors (R.D, J.Z, and F.W). If there are inconsistencies, the article is re-examined and discussed to reach an agreement.

### Summary measures and data analysis

Odds ratio (OR), weight mean difference (WMD), and standardized mean difference (SMD) presented with 95% confidence interval (CI) were used to pool analysis dichotomous and continuous variables, respectively. The difference was statistically significant when the *P* value was less than 0.05.

Using the Revman5.3 statistical software provided by the Cochrane collaboration network for statistical analysis. The heterogeneity of each study was tested by the *I*^2^ test. If *I*^2^ ≤ 50%, the statistical heterogeneity among the studies was small, and the fixed effect model was used to calculate the combined statistics. If *I*^2^ > 50%, it is suggested that there is heterogeneity among studies, and the causes of heterogeneity should be analyzed, such as whether there are significant differences in design, age, sex, body weight, type of stoma, and follow-up time. If no obvious clinical heterogeneity is found, the random effects model can be used for combined analysis, and the combined analysis results can be carefully explained. When necessary, sensitivity analysis was used to explore the source of heterogeneity among studies. Funnel chart was used to detect publication bias.

## Results

**Fig. 1:**
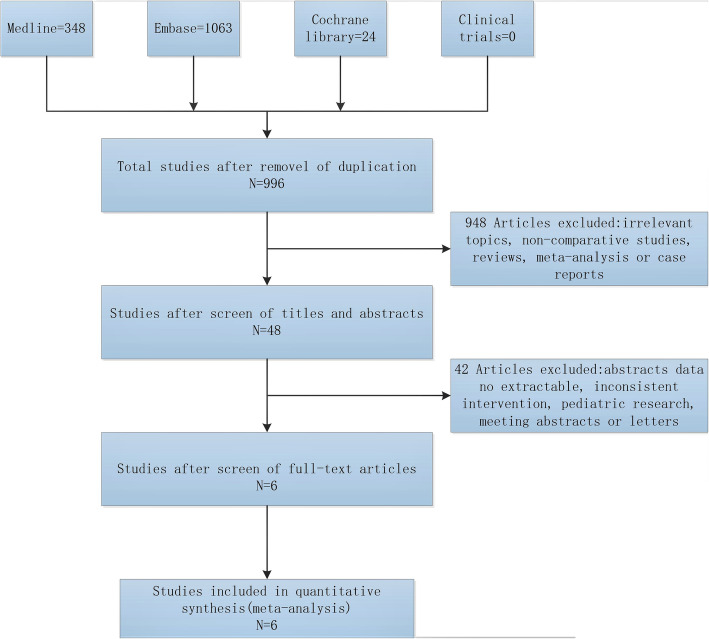
Flow diagram of the study selection

The search and screening process is shown in Fig. 1. A total of 1131 patients were included in 6 studies [9–14], including support rod group (*n* = 569) and non-support rod group (*n* = 562). All patients were followed up for more than 30 days. The basic characteristics of the study are shown in Table 1.

**Table 1:** Study and patient characteristics

Study	Year	Study type	Total patients	Sample size		Age		Gener(M/F)		BMI		The time of placement of the rod(d)	Stoma position	Follow-up time(d)
				rod	no rod	rod	no rod	rod	no rod	rod	no rod			
Speirs	2005	RCT	57	28	29	59 (IQR44–67)	63 (IQR52–70)	N/A	N/A	24	24	7	ileum	90
Heung-Kwon	2015	cohort study	32	20	12	58.4 (M45–82)	64.1 (M50–79)	8/4	14/6	21	20.6	28	ileum	90
Franklyn	2016	RCT	151	75	76	43.0 ± 15.8	44.7 ± 15.3	42/33	43/33	N/A	N/A	14	colon	90
Whiteley	2016	cohort study	515	260	255	59.7 (16.0)	64.6 (14.4)	154/106	150/105	N/A	N/A	12	ileum/colon	90
Uchino	2017	RCT	257	154	154	42.9 ± 15.2	41.9 ± 15.0	91/63	106/48	19.5	19.8	7	ileum	90
Zindel	2017	RCT	78	44	34	64.3 (SD10.5)	59.3 (SD12.3)	34/10	22/12	26.1	26.2	12	ileum	90

*RCT* randomized controlled trial, *IQR* quartile spacing, *SD* standard deviation, *M* median, *BMI* Body Mass Index

### Quality assessment of included studies

**Fig. 2:**
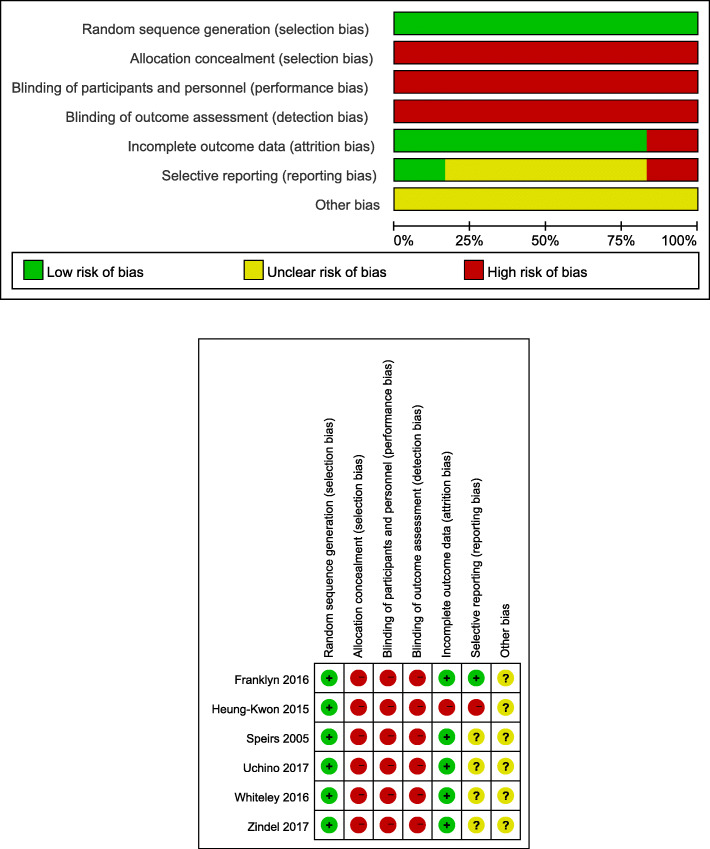
Risk of bias graph and risk of bias summary. Green +: low risk, red−: high risk, yellow: unclear risk

All the four RCT studies were randomly grouped by computer-generated random sequences, but because of the particularity of surgery, all studies did not use blind methods. The quality of 2 cohort studies was evaluated by the Newcastle-Ottawa scale, and the results showed that the score was 7, which was a high-quality study. Detailed quality evaluation is shown in Table 2. According to the bias risk assessment method recommended by the Cochrane system evaluator manual, the bias risk included in the study is shown in Fig. 2.

**Table 2:** NOS scale for cohort studies

Study	Selection				Comparability	Outcome assessment			Score
	1	2	3	4	5	6	7	8	
Heung-Kwon	1	1	1	1	0	1	1	1	7
Whiteley	1	1	1	1	0	1	1	1	7

According to the Newcastle-Ottawa scale: 1, the representativeness of the exposed cohort, 2, the choice of the non-exposed cohort, 3, the determination of the exposure, 4, no outcome to be studied in the cohort at the beginning of the study, 5, the control of confounding factors, 6, the determination of the outcome, 7, the follow-up time is long enough to observe the occurrence of the outcome, 8 sufficient follow-up rate

According to the Newcastle-Ottawa scale: 1, the representativeness of the exposed cohort; 2, the choice of the non-exposed cohort; 3, the determination of the exposure; 4, no outcome to be studied in the cohort at the beginning of the study; 5, the control of confounding factors; 6, the determination of the outcome; 7, the follow-up time is long enough to observe the occurrence of the outcome; 8, sufficient follow-up rate.

### Stoma retraction

**Fig. 3:**
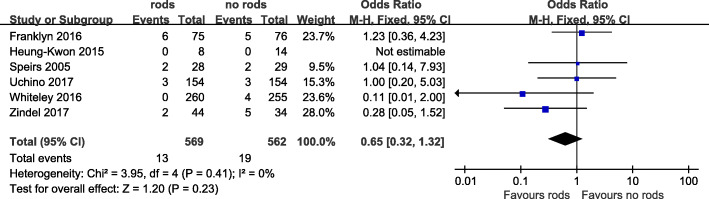
Forest plot of stoma retraction

The incidence of stoma retraction was statistically compared in 6 articles. A total of 32 cases of retraction occurred in a total of 1131 patients, with a total incidence of about 2.8%. The incidence was about 2.3% in the support rod group and 3.4% in the non-support rod group. The results of the meta-analysis showed that the support rod may reduce the risk of stoma retraction, but it has not reached statistical significance (OR = 0.65, 95% CI 0.32~1.32, *I*^2^ = 0%, *P* = 0.23) (Table 3, Fig. 3).

**Table 3:** Statistical results of stoma-related complications

Stoma-related complications	Name of the study^a^	Number of patients(n, events/total)		Statistical method, OR	Estimated effect, 95% CI	*P* value	I^2^ (%)
		rod	non-rod				
Stoma retraction	1、2、3、4、5、6	13/569	19/562	0.65	0.32 ~ 1.32	0.23	0
Stoma necrosis	1、5、6	26/342	4/319	6.41	2.22 ~ 18.55	0.0006	0
Peristomal dermatitis	4、5	121/414	57/409	2.93	2.01 ~ 4.27	< 0.00001	0
Mucocutaneous separation	1、5	23/335	11/331	2.14	1.03 ~ 4.47	0.04	0
Peristomal infection	1、5	5/335	2/331	2.53	0.49 ~ 13.18	0.27	0

*OR* odds ratio

^a^Name of the study: 1, Franklyn et al. [12]; 2, Oh et al. [10]; 3, Speirs et al. [9]; 4, Uchino et al. [14]; 5, Whiteley et al. [11]; 6, Zindel et al. [13]

### Stoma necrosis

**Fig. 4:**
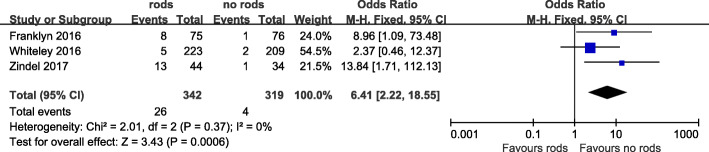
Forest plot of stoma necrosis

Three studies reported the incidence of stoma necrosis, including 342 cases in the bracing rod group and 319 cases in the unbracing rod group, and a total of 30 cases occurred in a total of 661 cases, including 26 in the bracing rod group and 4 in the unbracing rod group. The incidence rates were 7.6% in the bracing rod group and 1.2% in the unbracing rod group. The results showed that the necrotic rate of stoma in the bracing rod group was significantly higher than that in the unbracing rod group (OR = 6.41, 95% CI 2.22~18.55, *I*^2^ = 0%, *P* = 0.0006) (Table 3, Fig. 4).

### Peristomal dermatitis

**Fig. 5:**
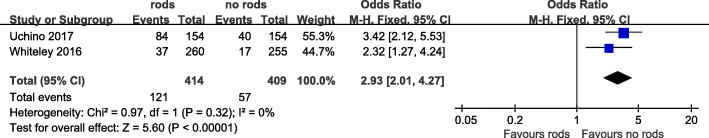
Forest plot of peristomal dermatitis

Two literature compared the incidence of peristomal dermatitis. The results showed that the incidence of peristomal dermatitis was higher, with a total of 21.6%, and the incidence of peristomal dermatitis in the stoma support rod group was significantly higher (29.2% vs 13.9%). The results of meta-analysis showed that the incidence of peristomal dermatitis was significantly increased in the support rod group, and the difference was statistically significant (OR = 2.93, 95% CI 2.01~4.27, *I*^2^ = 0%, *P* < 0.00001). There was no heterogeneity among the studies (Table 3, Fig. 5).

### Mucocutaneous separation

**Fig. 6:**
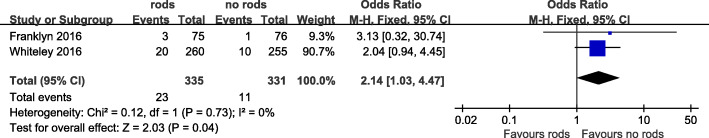
Forest plot of mucocutaneous separation

The difference in the incidence of mucocutaneous separation in stoma was reported in two studies. A total of 666 patients were included, and 34 cases of mucocutaneous separation occurred in the support rod group. The incidence in the support rod group was higher than that in the non-support rod group, the difference was statistically significant (OR = 2.14, 95% CI 1.03~4.47, *I*^2^ = 0%, *P* = 0.04), and there was no inter-study heterogeneity (*I*^2^ = 0) (Table 3, Fig. 6).

### Peristomal infection

**Fig. 7:**
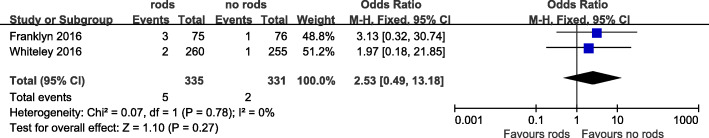
Forest plot of peristomal infection

Two studies recorded the incidence of peristomal infection. A total of 666 patients were included, of which 7 had peristomal infection. The results showed that the incidence of peristomal infection was low, about 1.1%, and there was no significant difference between the support rod group and the non-support rod group (OR = 2.53, 95% CI 0.49~13.18, *I*^2^ = 0%, *P* = 0.27) (Table 3, Fig. 7).

### Other complications

The study of Whiteley et al. [11] analyzed the difference in the incidence of skin irritation in enterostomy, and the results showed that the total incidence was about 10.5%. The incidence in the support rod group was significantly higher than that in the non-support rod group. Oh et al. [10] reported that more patients in the support rod group said it was difficult to change their pockets (72.7% vs 14.3%, *P* = 0.002). Speirs et al.’s study [9] analyzed the state of stoma activity, and the results showed that 83% of patients in the non-bracing rod had a more active stoma, compared with 79% in the bracing rod group, and the difference was not statistically significant.

### The length of placement of stoma rod and hospital stay

It was not specified length of the rod that has been placed in the study of Zindel et al. [13]. The duration of rod use was short being only 3.5 days in the study of Whiteley et al. [11], but in another two studies [9, 14], the rod was removed on the 7–8th day after surgery, while in the RCT performed by Franklyn et al. [12], the rod was left in place until the 10–14th day after surgery, and even more, according to the study of Oh et al. [10], the rod was braced until the 4–28th day after surgery.

**Fig. 8:**
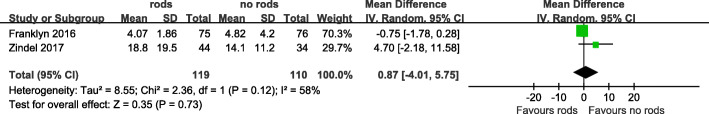
Forest plot of the length of stay

Three studies reported length of stay, and sufficient data was available in two of three studies for inclusion in the meta-analysis. There was no significant difference in hospital stay between patients that did and did not have a stoma rod (WMD = 0.87, 95% CI − 4.01~5.75, *I*^2^ = 58%, *P* = 0.73) (Fig. 8)

### Analysis of heterogeneity and publication bias

**Fig. 9:**
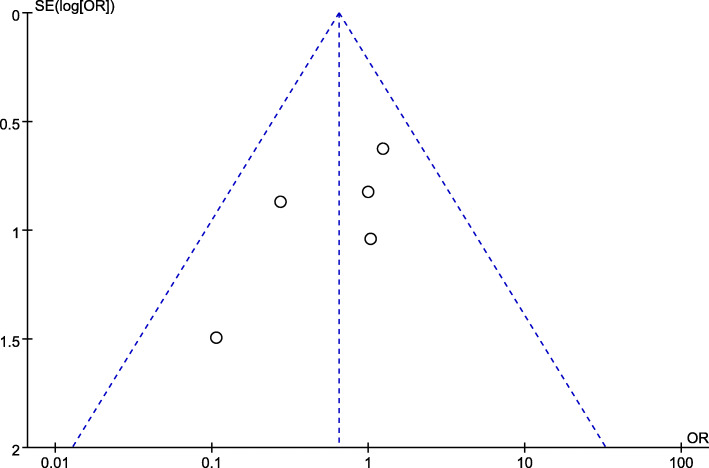
Funnel plots of stoma retraction

Because all the studies included in this paper have the same research purpose, have high methodological quality, and *I*^2^ is zero in the analysis of most outcome index, considering the homogeneity among the studies, there is no need for inter-study heterogeneity analysis. Taking the incidence of stoma retraction as an example, no significant publication bias was observed in the funnel plots of it (Fig. 9).

## Discussion

### Stoma rods and stoma retraction

At present, the details of the use of stoma support rods are not unified in different countries and regions, and there are all kinds of support rods, especially for the placement time, there is still no conclusion. It is reported that the retention time of the support rods in different medical centers is different; most of which are placed for 5 to 7 days [15–17], some for 7 to 14 days [18, 19], or even 3 to 4 weeks [20, 21] later. There is currently no literature to show the effect of placement time of support rods on the incidence of stoma retraction.

It was reported that the incidence of stoma retraction was about 0 to 1.4% [22–25], and support rods were routinely used in these studies. In contrast, some studies have reported a high retraction rate of stoma, about 5 to 26% [20, 26, 27], or even as high as 40% [28]. However, there are great differences in the population of these studies, some only include ileostomy, or some only include colostomy, which may be an important reason for the difference in statistical data. Besides, the definition of retraction is not unique. Some studies define stoma mucosa below the skin as retraction [5], while other studies require mucosa to be at least 0.5 cm below the skin surface to diagnose retraction [29], so different definitions used in different studies may also lead to differences in the incidence of stoma retraction. Nowadays, this meta-analysis showed that of the 1131 patients included, 32 had stoma retraction (2.8%), the overall contraction rate was very low, and there was no statistical difference in the incidence of stoma retraction between the rod group and the non-rod group.

And meanwhile, an observational cohort study of Whiteley et al. [11] showed that 91.4% of enterostomies used support rods in 2003, while in 2012, only 10% of enterostomies used rods to prevent retraction, and in the process of significant reduction in the use of stoma support bars, the incidence of retraction did not change significantly, which also proved from the side that the occurrence of stoma retraction was not significantly related to the use of support rods.

In summary, we think that although some surgeons are still keen to use support rods to prevent retraction, given that this meta-analysis shows that it does not reduce the rate of stoma retraction, and even adding a device that increases complications [30, 31]. Thus, it is an unfounded surgical dogma to worry about increasing the incidence of stoma retraction without rods, we do not recommend routine use of support rods to prevent stoma retraction.

### Stoma rods and stoma necrosis

Inadequate dissociation of the tube, too short and tensioned stoma tube, intra-abdominal inflammation, obesity, scar adhesion, and short mesentery may be all related to stoma retraction, and it is reported that stoma retraction can lead to stoma necrosis [28], which is directly caused by the increase of enterostomy tension caused by poor enterostomy position or lack of intestinal motility [21, 32], it is also reported that the existence of high BMI is also related [33]. However, the application of support rod cannot reduce the occurrence of these problems; the results of this meta-analysis showed that the incidence of stoma retraction in the bracing rod group was not significantly lower than that in the non-supporting rod group, and the difference was not statistically significant (*P* = 0.23), so the support rods cannot reduce the occurrence of stoma necrosis from the sources, but only provides the resistance against retraction in the early stage of enterostomy. Moreover, the use of support rods is bound to oppress the surrounding skin, increase mesangial tension, may aggravate ischemic necrosis, and affect stoma nursing.

Also, certain patient factors, such as long-term use of steroids, malnutrition, diabetes, and smoking may lead to poor wound healing and/or peristoma infection [34], leading to stoma retraction, and further aggravating stoma necrosis. However, among the 6 articles included, the relevant data are not comprehensive, and many of them have no routine records, so they cannot be analyzed. Besides, the extremely low incidence of stoma retraction makes effective analysis and meaningful interpretation very difficult.

### Peristomal dermatitis, peristomal infection, and mucocutaneous separation

Adequate sealing of the skin around the enterostomy can prevent fecal leakage and reduce subsequent skin erosion and pain around the enterostomy [19]. In one study, which compared the effects of skin-level stoma support rods and subcutaneous support rods on skin irritation around the stoma, it reported that due to persistent fecal leakage, the skin-level support rod group had 70% of the surrounding skin irritation, and it was only 6% [33] in the subcutaneous support rod group. It is worth noting that in our study, the incidence of peristomal dermatitis in the unsupported group also decreased significantly, and the difference was statistically significant. The risks of other theories reported in the literature for the use of stoma support rods may include stress ulcers or skin necrosis caused by continuous pressure of the support rods [20, 27], peristoma infections caused by fecal contamination of the support rods passing through the mesenteric margin [25, 34], mesenteric ulcers, and even stoma necrosis [33]. Since the support rod is indeed related to complications [35] (such as peristoma dermatitis, peristomal infection, mucocutaneous separation, and even stoma necrosis), which will have a great impact on patients’ nursing and quality of life, it is not recommended for regular use.

Also, the insertion of stoma support rods during operation can directly lead to accidental surgical complications (intestinal wall injury and perforation during insertion). Some studies also suggested that the application of a support rod can increase the incidence of intestinal obstruction after loop stoma. However, in this systematic review, most of the patients in one study (> 90%) had the exhaust on the third day after the operation, and the stoma was functioning well.

### BMI and stoma retraction

The stoma-specific morbidity score (SSMS) of 78 patients were evaluated by Zindel et al. [13]. The results showed that a high body mass index (BMI) was significantly correlated with high SSMS, but no difference based on the presence of a stoma rod. The median BMI of the study population is 26 kg/m^2^; in univariate analysis, the relative risk of people with higher BMI (> 26 kg/m^2^) increased fivefold. The relative risk of SSMS > 3 was 5 (*P* < 0. 01), and the relative risk of SSMS > 4 was 4.8 (*P* = 0.025). The correlation was still significant when adjusting for age, diabetes, operation time, and a randomized multivariate model (*n* = 65) (*P* = 0.02). The reason may be that people with high BMI have thicker subcutaneous layers and relatively shortened mesentery, which may lead to greater tension in abdominal wall stoma, reduce blood supply to abdominal wall and stoma, and increase the risk of retraction and/or necrosis.

However, other studies have shown that BMI itself is not a factor affecting the incidence of stoma retraction [18, 36]. Considering that a large proportion of patients are overweight or obese [16, 29, 37, 38], this paper also analyzed the effect of BMI on stoma retraction, but our study did not find that 32 patients with stoma retraction had obvious characteristics on BMI, which indicated that there may be no significant relationship between high BMI and the incidence of stoma retraction. And meanwhile, there were two included studies reported that the incidence of stoma retraction was also low in patients with low BMI, and the support rod had no significant effect on the incidence of stoma retraction. From this, we may conclude that the effect of support rods on the incidence of stoma retraction in people with different BMI is not significant.

### Limitations

This meta-analysis has the following limitations: (1) only 6 articles are included in this paper, and the number of studies is small, and some of them may have the problem of report bias; (2) due to the inconsistent reporting results and some of the results do not provide original information, some outcome indicators cannot be analyzed by meta. As the meta-analysis is a secondary study, it is greatly affected by the quality of the original literature included in the analysis, and there may be some limitations such as bias in the evaluation process. In the future clinical application, more multicenter, large sample, and high-quality evaluation of the intervention effect of stoma support rod use is needed, and more evaluation indicators are included to provide more reliable evidence.

## Conclusions

In this study, through the analysis of the application value of the support rods, the results show that: (1) the incidence of stoma retraction is low, and the application of support rods cannot significantly reduce the risk of retraction. (2) The application of support rods will lead to more complications after loop enterostomy (such as peristoma dermatitis, peristomal infection, mucocutaneous separation, and even stoma necrosis). It has a great impact on the nursing care and quality of life of patients. To sum up, it is an unfounded surgical dogma to worry about increasing the incidence of stoma retraction without support rods. With correct surgical technique, a loop stoma can be made to pout without the need for a supporting rod for all patients, including obese patients; we do not recommend the routine use of stoma support rods.

## Data Availability

Data sharing is not applicable to this article as no datasets were generated or analyzed during the current study.

## Abbreviations

RCTs: Randomized controlled trials
BMI: Body mass index
WMD: Weighted mean difference
Cl: Confidence interval
OR: Odds ratio
